# Preparation, Characterization, and In Vitro Sustained Release Profile of Resveratrol-Loaded Silica Aerogel

**DOI:** 10.3390/molecules25122752

**Published:** 2020-06-15

**Authors:** Lili Qin, Yiwei He, Xinyu Zhao, Ting Zhang, Yao Qin, Ai Du

**Affiliations:** 1 Sports and Health Research Center, Department of Physical Education, Tongji University, Shanghai 200092, China; 1831832@tongji.edu.cn (Y.H.); 1831833@tongji.edu.cn (X.Z.); 1931799@tongji.edu.cn (Y.Q.); 2 School of Physics Science and Engineering, Tongji University, Shanghai 200092, China; 1730965@tongji.edu.cn

**Keywords:** resveratrol, silica aerogel, drug carrier, characterization, release kinetics

## Abstract

Silica aerogel, a kind of nanoporous material, is regarded as a desired drug carrier for its low toxicity, high specific surface area, and excellent biocompatibility. Using silica aerogel in a drug carrier may be an appropriate method to improve the performance of pure resveratrol. In this study, resveratrol-loaded silica aerogel (RSA) as a drug delivery system was prepared by the sol-gel method. Before gelling, resveratrol was added into the hydrolyzed tetraethyl orthosilicate (TEOS) ethanol solution then dispersed by stir and ultrasound. The results showed that RSA has a high loading rate of 19% with low cost and excellent biocompatibility. The SEM images showed that silica aerogel wraps up outside the resveratrol. Sustained releasing effect could be observed in RSA after 1 h, while pure resveratrol did not display this. The release of RSA lasted for over 6 h, and the release amount reached over 90% and 80% in either simulated gastric fluid (pH = 2.0) or phosphate-buffered saline (pH = 7.4) at 37 °C. Preliminary in vitro toxicity test revealed RSA to be biocompatible and stable; and when coupled with the anti-inflammatory effects of resveratrol, showed good potential for osteoarthritis treatment.

## 1. Introduction

Osteoarthritis is a prevalent and complicated disease, where patients often suffer from pain and discomfort. It is estimated that by 2032, the number of people over the age of 45 with osteoarthritis will rise from 25% to 29% [1] with peaks of incidence generally around the age of 75 [2]. This is followed by an increase in medical expenses; according to estimations, the medical cost for osteoarthritis (OA) patients will account for nearly 2% of the gross domestic product (GDP) in middle- and high-income countries.

Currently, oral administration is still considered as the most commonly used treatment [3]. Resveratrol has been considered to have the potential to alleviate or even cure osteoarthritis [4,5]. However, the physical properties of resveratrol, including metabolic inactivation, insolubility in water, and instability, limit its application as a treatment for osteoarthritis [6]. At the same time, the treatment of osteoarthritis is a long-term chronic process, which requires that the drug possess the controlled release property [7]. With the development of nano-materials for drug delivery systems, the application fields of a series of drugs have been widely expanded [8,9,10]. This gives a considerable prospect for improving the clinical application of resveratrol.

Silica aerogel is a promising nano-porous material for drug delivery systems, mainly due to its high loading capacity, excellent adsorption properties, and high biocompatibility [11,12,13,14]. In addition, silica aerogel has a mature preparation process and can be modified by the manipulation of synthetic conditions in simple approaches [15]. In the past ten years, a series of researchers have been working on aerogels as agriculture drug carriers [16,17] or even in veterinary medicine [18]. Through supercritical drying, some insoluble drugs such as ibuprofen and ketoprofen can be loaded, and a high drug loading rate (10%–30%) obtained [19,20,21,22]. Controlled release of a loaded drug is an essential property for a drug delivery system [23,24,25]. Mesoporous silica (MCM-41) was first used as a sustained drug delivery system; in vitro release studies showed that drugs loaded on silica aerogel could achieve sustained release effect in the long-term release process [26]. The dissolution of drugs is limited by the skeleton structure of silica aerogel, so that the release rate decreases with the prolongation of release time [27]. Therefore, with the characteristics of controlled release and high loading capacity, silica aerogels are suitable for loading resveratrol in treating osteoarthritis.

Drugs loading can be achieved by two different processes, namely adding drugs in the sol-gel process or in the post-processing of synthetic aerogels [28]. The most commonly used approaches are based on diffusion into the wet gel before drying, including the supercritical fluid loading method [10]. However, from the perspective of clinical application, absorption in supercritical fluid has the problems of a complex process, high cost, and low production efficiency; while the way of loading drugs before gelation is relatively simple and flexible for various target compounds [28,29], and thus may be more suitable for the pharmaceutical industry.

**Figure 1:**
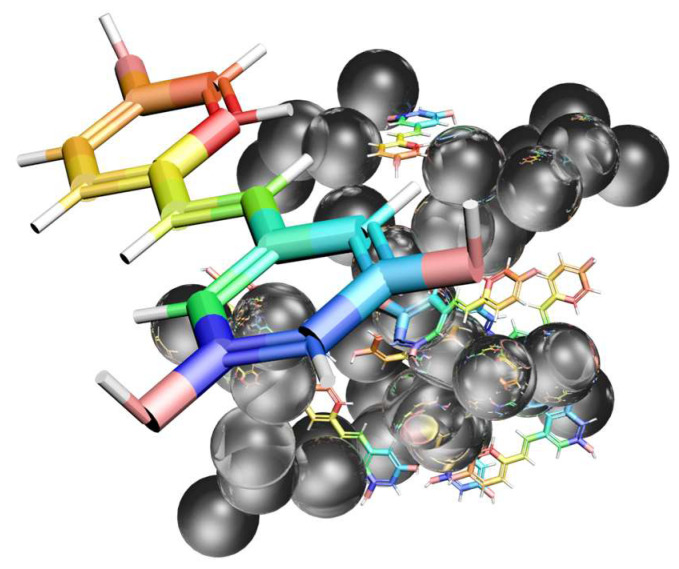
Diagram of resveratrol adsorption on silica aerogel.

In this study we prepared the resveratrol-loaded silica aerogel (RSA) by loading the drug during the pre-gel process. A schematic diagram (Figure 1) indicates the structure of resveratrol-loaded silica aerogel. Physical adsorption and coating forms existed simultaneously in the RSA, leading to good release performance with a sustained released period of over 6 h. In vitro release experiments were conducted to study the release behavior of simulated gastric fluid (pH = 2.0) and phosphate-buffered solution (pH = 7.4). Moreover, the cytotoxicity effect of RSA was evaluated on TC-28a2 human cartilage cells. This study suggests that RSA has potential as osteoarthritis medication.

## 2. Results

**Figure 2:**
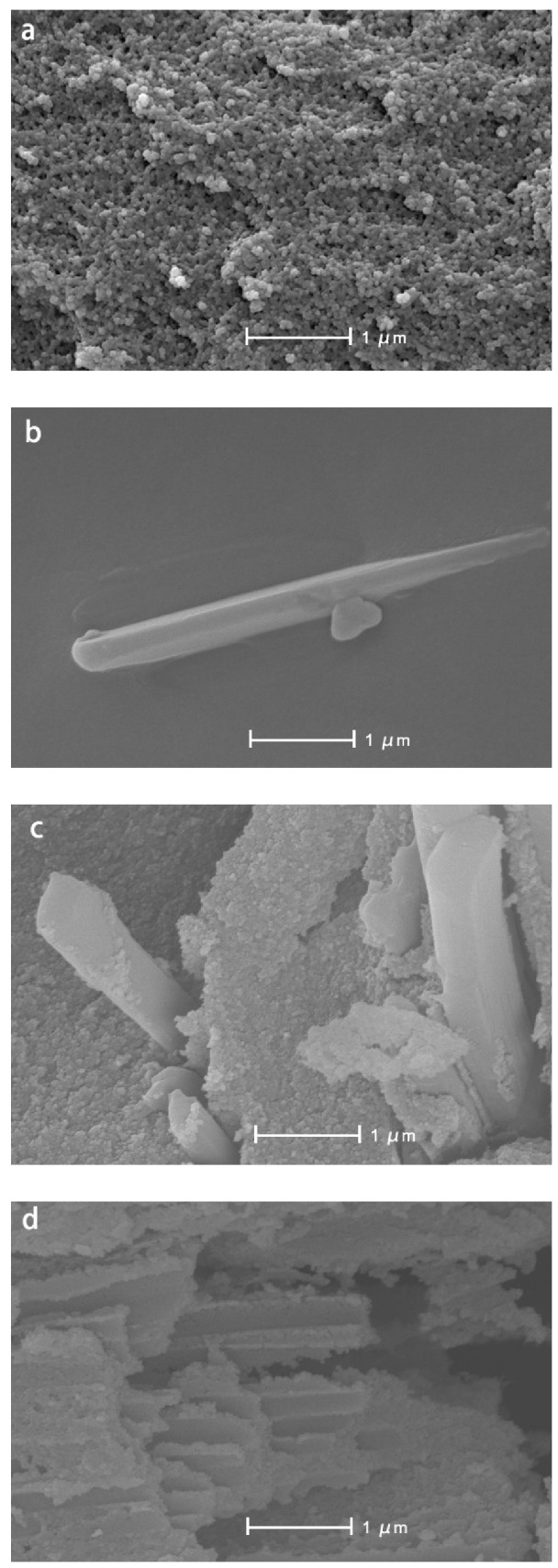
SEM images of silica aerogel (**a**), resveratrol (**b**), and resveratrol-loaded silica aerogel (**c**,**d**).

The microstructure of silica aerogel was captured by SEM (Figure 2). The samples have a typical nano porous structure, and the skeleton particles are loose and evenly distributed. In Figure 2a, the silica aerogel possesses a tight network of interconnected nanostructures. In Figure 2b, the resveratrol shows a sheet-like structure and a distinct geometric edge. This is normal for freeze-dried drug powders. The cross-section of RSA is shown in Figure 2c. Resveratrol can be seen coated by silica aerogel. Figure 2d shows the structure of the RSA after calcinating in 500 °C. The sheet-like structure of resveratrol disappeared during calcination, while the regular edge of the silica aerogel structure out of resveratrol was retained.

**Figure 3:**
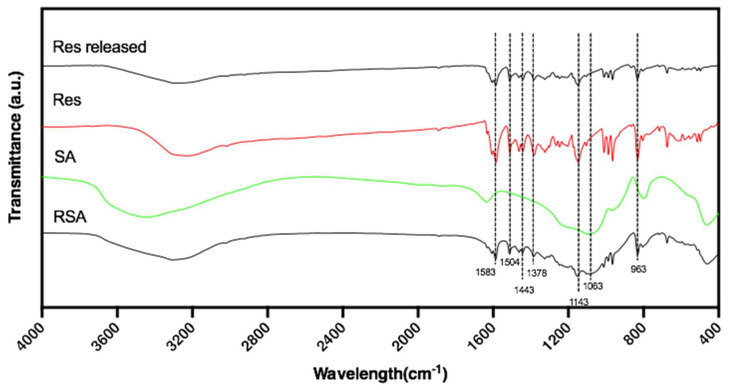
FTIR spectra of resveratrol-loaded silica aerogel (RSA), resveratrol (Res), silica aerogel (SA), and Res released from RSA in phosphate-buffered saline (PBS) (Res released).

The FTIR spectra of resveratrol, silica aerogel, and RSA are represented in Figure 3. The FTIR spectrum of resveratrol showed the presence of the following main peaks. The peak near 3200 cm^−1^ was assigned to the free O–H stretching vibration. A double peak at 1600 cm^−1^ and 1583 cm^−1^ corresponded to C–C aromatic double bond stretching and C–C olefinic stretching. In addition, the peaks at 1504 cm^−1^ and 1443 cm^−1^ reflected the benzene skeleton vibrations and C–C stretching vibration as evidenced by the peak at 1378 cm^−1^. The peaks at 1063 cm^−1^ and 963 cm^−1^ were ascribed to the bending vibration of C=C–H, which demonstrated the transformation of resveratrol [30,31]. The spectrum of silica aerogel showed peaks at 1200–1000 cm^−1^ which expressed the Si–O tetrahedron symmetrical vibration peak with strong covalent bond vibrations of silicon-oxygen. This wavenumber region reveals the presence of a silica network (Si–O–Si) [32]. After the in vitro drug release test, we filtered and dried the solution to obtain the released resveratrol powder. FTIR spectrum of the resveratrol released from RSA in phosphate-buffered saline (PBS) was tested. Compared to the pure powder of resveratrol, the characteristic peaks on the FTIR spectra of resveratrol did not change, which can prove that the chemical structure of resveratrol was retained.

**Figure 4:**
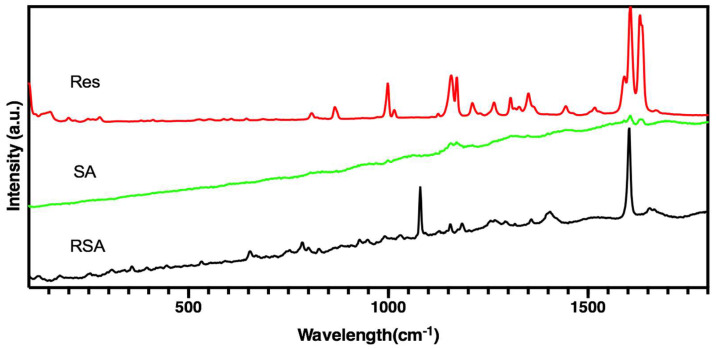
Raman spectra of silica aerogel, resveratrol, and resveratrol-loaded silica aerogel.

**Figure 5:**
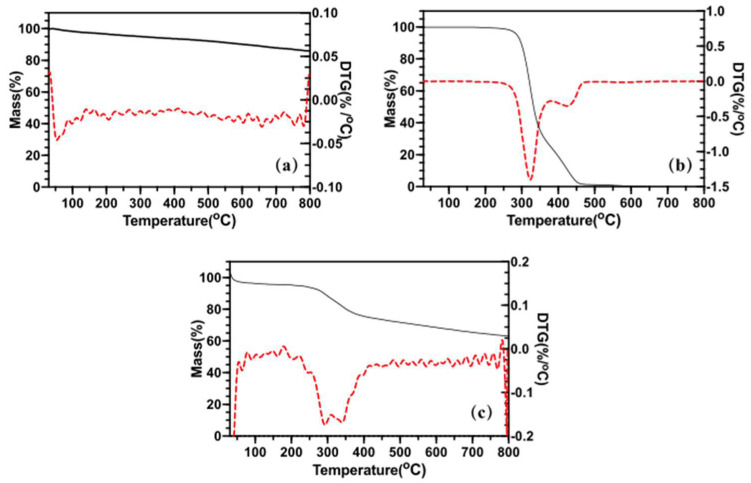
TG–DTG curve of silica aerogel (**a**), pure resveratrol (**b**), and RSA (**c**).

Figure 4 shows the Raman spectra of three samples. The prominent feature of resveratrol was two strong bands at 1633 cm^−1^ and 1604 cm^−1^. Both bands assigned to a combination of ν(C=C) and δ(C–H) vibrations of the trans-olefin carbons together with ν(C=C) vibrations of the phenyl rings, whereas the band at 1589 cm^−1^ was due to ν(C=C) associated with δ(O–H) vibrations [33]. A typical room temperature Raman spectrum of silica aerogel is shown in Figure 5. The obvious slope of the background between 500 and 1700 cm^−1^ may be attributed to fluorescence [34]. In the spectrum of RSA, weak intensity peaks can be observed nearly 490, 605, 810, and 970 cm^−1^ related to the Si–O–Si network [35].

From the FTIR spectrum and Raman spectrum of three samples in Figure 3 and Figure 4, the spectrum of RSA contained all the characteristic peaks in both resveratrol and silica aerogel that demonstrate resveratrol existed in the RSA. Compared with pure drugs in their crystalline form, all peaks were detected in the same position. These results indicate that the drug remained intact during preparation and maintained its structure. Furthermore, no additional characteristic peaks appeared, indicating that there was no obvious interaction between the drug and the macrocyclic.

Figure 5a shows the nearly 10% weight loss of pure SiO_2_ aerogels at 800 °C. Theoretically, SiO_2_ is a stable inorganic substance, and weight loss of silica aerogel should not be detected. It is presumed that the residual ethanol, desorption of water and condensation of surface hydroxyl and surface alkoxy groups are the main reasons. From Figure 5b, the DTG curve shows the losing weight rate of resveratrol. Most of the resveratrol was burned at 450 °C. Figure 5c shows the weight loss of RSA. The loading rate was equal to the difference between the weight loss of RSA and pure silica aerogel. Resveratrol was completely calcined at nearly 450 °C. Considering the 5% weight loss of silica aerogel at 450 °C, the loading ratio of RSA could be calculated as 19% according to Equation (1).

**Figure 6:**
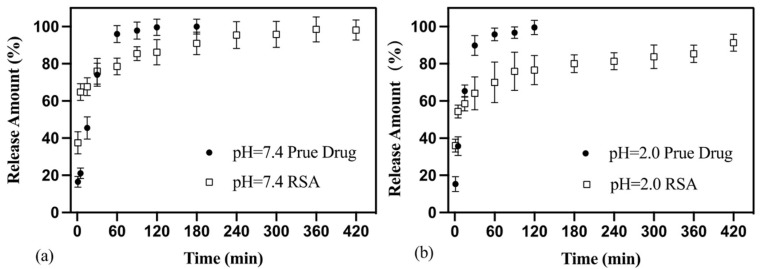
Release data of RSA and pure drug in phosphate-buffered solution (pH = 7.4) (**a**) and simulated gastric fluid (pH = 2.0) (**b**) at 37 °C.

The release curves of pure drug powder and RSA are shown in Figure 6. Over 90% of resveratrol powder released rapidly in 1 h in both simulated gastric fluid (pH = 2.0) and phosphate-buffered solution (pH = 7.4) conditions. The release rate was close to a fixed value and the release curve was close to a straight line. Between 1 h and 3 h, the concentration of released resveratrol was almost unchanged which suggests that resveratrol completely released into the solution.

As for RSA, the duration of the release phase could be divided into two phases. In the first 30 min, drug release concentrations increased rapidly; about 60% of the resveratrol released into the solution at 30 min. The release speed of resveratrol from RSA was faster than pure powder. After 30 min, drug release entered the second phase. Compared to the first phase, the rate of drug release slowed significantly. With the prolongation of dissolution time, the slope of each release curve in the graph decreased. The concentration of resveratrol rose robustly until approaching the theoretical maximum concentration; this stage could last more than 6 h.

**Figure 7:**
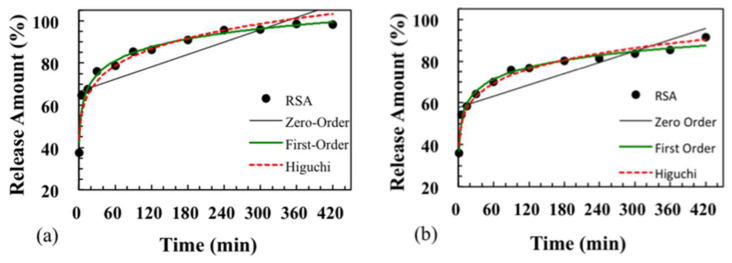
Fitting of released data of RSA in phosphate-buffered solution (pH = 7.4) (**a**) and simulated gastric fluid (pH = 2.0) (**b**) at 37 °C to three different models: zero-order, first-order, and Higuchi model.

Three types of dissolution-diffusion kinetic models including zero-order, first-order, and Higuchi model were used to explain the released mechanism of RSA and pure resveratrol powder. Using regression analysis, the fitted curves are shown in Figure 7, and correlation coefficients (R^2^) of the regression equation are listed in Table 1. In different in vitro solution environments, the trend of the released curve was almost the same for RSA. The pure drug was more suitable to be explained by the zero-order model in the first 30 min, with R^2^ = 0.995 (pH = 7.4) and R^2^ = 0.999 (pH = 2.0). The Higuchi model and first-model were not suitable for the pure drug release pattern. The release of resveratrol from silica aerogel fit the first-order and Higuchi models much better.

**Figure 8:**
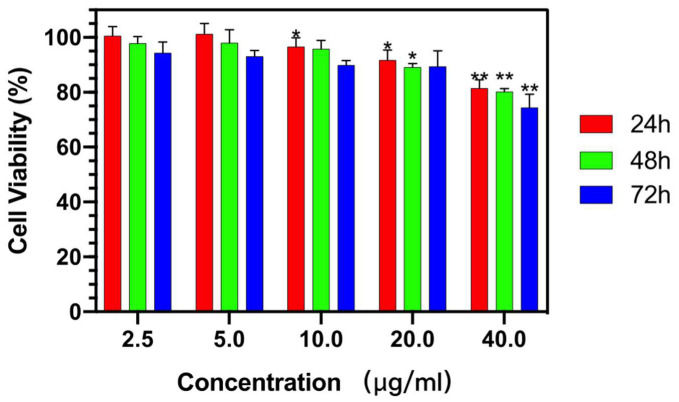
In vitro cell viability analysis of RSA treated for 24, 48 or 72 h at concentrations of 2.5, 5, 10, 20, and 40 μg/mL by MTT assay. Results represent the means of three independent experiments and error bars represent the standard error of the mean. (* *p* < 0.05, ** *p* < 0.01).

The cell viability of RSA was tested in cartilage cells TC-28a2 by MTT assay. The yellow tetrazolium MTT was reduced by metabolically active cells, in part by the action of hydrogenase enzymes to generate reducing equivalents such as NADH and NADPH. The resulting intracellular purple formazan was solubilized and quantized by spectrophotometric means. As shown in Figure 8, the RSA did not show an obvious toxicity owing to the fact that the rate of living cells was maintained at 90% after incubation for 72 h at each of the tested concentrations below 20 µg/mL. Even when the concentration reached 40 µg/mL, the relative cell viability remained at about 80% after 72 h treatment to TC-28a2 cells.

## 3. Discussion

In this study, RSA was successfully prepared by loading the drug in a more convenient way through pre-gel loading and freeze-drying, which improve the efficiency of preparation while saving cost. As for clinical application, the cost and efficiency of preparation are necessary to consider. Compared to the supercritical method for loading drug, the pre-gel way is more convenient and time-saving, eliminating the path of supercritical loading drug and impregnated loading drug during solvent replacement. Through the supercritical method the total preparation period of RSA was reduced from more than one week [36] to 4 days. Without supercritical equipment, the preparation cost was reduced, and the yield of the RSA was increased. All these satisfy the demand for clinical application. When the drug was put into the solution of the precursor, resveratrol was freely dispersed in the solution through dissolution. During gelation, the formation of a silicon oxide network separates resveratrol and limits it in the network. In Figure 2d, the structure of silicon dioxide remains the basic characterization. The smooth geometry of resveratrol, an indication that its loading in silica aerogel was calcined, was no longer visible. This proved that resveratrol was carried on silica aerogel. The pure resveratrol powder had a large-scale irregular geometry structure. Through the method of drug loading before gelation, resveratrol was separated by a silica skeleton network and formed into a rod-like structure with a radius of several hundred nanometers.

Compared with other research on loaded drugs similar to resveratrol (25%, 28%) [37,38], the loading rate of RSA (19%) is relatively low. This might be because some of the drugs diffuse from the inside due to the concentration difference during the post-gel solvent replacement process, which is obviously in the hydrogel. With the prolongation of the solvent replacement period, the drug diffuses out and the amount of the drug in the aerogel drops.

The structures of RSA help to prevent the drug from being exposed to the outside and protect its stability [19]. Different from the traditional system through diffusion-adsorption mechanism loading drug, in this study the form of prepared RSA was small particles of about a few microns. Resveratrol was coated on the silica aerogel rather than adsorbed into the silica skeleton. Both the surface and the outer edge of RSA were coated with silicon oxide. Different from the coating structure formed by spraying a layer of material outside the drug [22], a small cross-section was in contact with the outside. In its cross-section, a rod-like resveratrol geometric edge can be seen. The dissolution of the drug through the cross-section is limited.

Due to the simple crystal structure, the resveratrol release from RSA burst in water and was much faster than pure drug in the first 30 min (Figure 6). For the case of the crystalline drug, it took a process of destruction before the drug dissolved in the solution [37]. The release duration increases with the enlargement of microspheres’ particle size. However, the diameter of resveratrol loading on RSA was about hundreds of nanometers. The solution was more likely to penetrate the exposed section of the drug and released drug directly. While part of drug loading was on the aerogel, this step was eliminated, and the dissolution process was accelerated [38].

Both released rates of RSA in phosphate-buffered solution and simulated gastric fluid are far slower than the pure drugs. The dissolution curves of RSA tend to be smooth and equilibrium in the second phase from 1 h to 6 h. It can be inferred that the release time of RSA sustained more than 6 h. At this time, the speed of drug release was limited by the speed of solution infiltration into the outer layer of the silicon aerogel. The main reason for this limitation is the mesoporous material in silica aerogel and the dissolution becomes difficult, which leads to a decreased dissolution rate. Meanwhile, in the deeper layer, the drug was difficult to release because it was far away from the cross-section exposed to the outside. In addition, both sides were coated with silica aerogel, which can prolong the release time of drugs without decreasing the efficiency.

Since the release amount of pure drug was less and the concentration reaches a plateau when the release time is more than 30 min, the first 30 min should be selected to reflect the real release pattern. The results (Table 1) show that the release of pure drug is similar to that of rapid drug release following the zero-order model. The release curve of RSA followed the other two models much better, with R^2^ much higher than 0.9 for both the first-order model and Higuchi model. The first-order model can be used to characterize the release rate of pharmaceutical dosage forms in porous media. It reflects that the drug release rate changes with the concentration, without considering the shape, size, and internal structure of the drug carrier. Drugs in solution are more likely to diffuse based on Fick’s law. Higuchi model reflects drug release from a porous system without swelling or contracting, and the diffusion rate of the solvent decreases with the increase of the path in the structure.

Virtually, two forms of drug loading on the silica aerogel exist simultaneously: drug loading on aerogel surface partly exposed to external solution and drug contacting with aerogels in the three-dimensional network structure. Thus, the real diffusion of drugs comes from three aspects: the scattered drug powder, the drug coated in silica aerogel and the drug at the lateral edge of RSA. Therefore, the drug release may be a co-effect, including drug desorption from silica and drug release from the coating. Drug release from RSA is more similar to the first-order model R^2^ = 0.974 (pH = 7.4), R^2^ = 0.985 (pH = 2.0), which means the major mechanism for resveratrol release from the coating silica aerogel plays a major role in RSA. Obtained results show that the impregnation of active pharmaceutical ingredients could reduce the contact area between pure drug and solution, decelerating the release time from the aerogel and providing a controlled release effect. Aerogels as carriers for active drugs have a positive effect on the pharmacokinetic properties of the drug substance.

The biological cytotoxicity of RSA was tested by MTT assay in cartilage cells, which will be further studied for their treatment effect of osteoarthritis. In Figure 7, the cell survival rate maintained a minor toxicity to the cells with the increasing drug concentration after incubating for 24 h, 48 h, and 72 h. Moreover, for all concentrations of the RSA, the cell viability in the first 24 h almost remained at 90%. The high cell viability of RSA was closed to the biogenic silica nanoparticles which showed excellent biocompatibility [39]. The results indicate that RSA cannot cause significant cell cytotoxicity and has potential to serve as a safe drug delivery system in the cellular system. In addition, incorporating resveratrol with silica aerogel dispersing in the aqueous phase should facilitate drug release that is better sustained. It also proves that silica aerogel is a noncytotoxic, biocompatible, and safe vector for loading resveratrol, and might be able to deliver therapeutics in a dose-specific manner.

## 4. Materials and Methods

### 4.1. Material

Tetraethyl orthosilicate (TEOS) was supplied by Sinopharm Chemical Reagent Co., Ltd. (Shanghai, China). Resveratrol (Res), 3,4′5-Trihydroxy-trans-stilbene, was purchased from Aladdin (Shanghai, China). Its content was over 99%, as reported by its supplier. Phosphate-buffered saline (PBS) and simulated gastric fluid (ChP) were purchased from Scientific Phygene (Fuzhou, China). All other chemicals were obtained commercially as analytical-grade reagents.

### 4.2. Preparation of the Silica Aerogel

We used TEOS as the precursor. Silica aerogel was produced by the two-step method through the sol-gel process. First, TEOS was mixed with ethanol, deionized water, and hydrofluoric acid (HF). The volume ratio of each substance was TEOS:EtOH:H_2_O:HF = 6:12:1:0.018. The mixture was stirred for 30 min at a stirring speed of 500 rpm. After gel formation samples were aged for two days and impurities were displaced by solvent displacement, mainly hydrofluoric acid. Finally, the gel was quickly frozen and then freeze-dried for 24 h to obtain the pure silica aerogel.

### 4.3. Preparation of the Resveratrol-Loaded Silica Aerogel (RSA)

Resveratrol was selected for loading on silica aerogels. After preparation of TEOS ethanol solution, powder of resveratrol (50 mg) was added into the precursor (25 mL) and ultrasonic dispersed for 30 min and then stirred for 30 min at 500 rpm. Hydrofluoric acid was added to make it a gel. This was followed by gelation and aging. Sufficient deionized water was used for solvent replacement. The solvent was replaced three times, and the interval of each time did exceed 1 h. Then the process of freeze-drying was the same as preparation of the silica aerogel. Using this method, the drug molecules dissolved in the initial solution that were expected to be trapped in the aerogel network during gelation.

### 4.4. Characterization of Material

#### 4.4.1. Surface Morphology Analysis

The morphologies of silica aerogel, resveratrol, and RSA were observed through SEM. SEM image was observed by a Field Emission Scanning Electron Microscope (S-4800, Hitachi, Tokyo, Japan). We also tested the powder of RSA calcined at 500 °C for 30 min. At this temperature, organic matter such as resveratrol tends to decompose or carbonize, while silicon dioxide does not. All the samples were grinded into powder and plated gold on the surface. The magnification of the SEM image was 40,000×.

#### 4.4.2. FTIR Analysis

Fourier Transform In Frared Spectrometer (FTIR) analysis was performed on a Nicolet 560 FTIR spectrometer (Nicolet, Madison, USA). Infrared spectra were studied by the KBr tablet method. Resveratrol, silica aerogel, and resveratrol-loaded silica aerogel (RSA) were grinded into powder, mixed with potassium bromide, and then compressed as a KBr tablet. All spectra were scanned from 4000 to 500 cm^−1^ at a resolution of 2 cm^−1^. The background spectrum was obtained in air. Data processing included compensation for water and carbon dioxide atmosphere.

#### 4.4.3. Raman Analysis

Raman spectroscopy was used to characterize the structural electronic properties, crystal structure, and disorders in silica aerogel (SA) and RSA; it was also used to measure the induced structural changes during the preparation process. Raman spectra of samples were registered in solid state or powder with a Renishaw Raman RM2000 instrument, which was equipped with an electrically cooled Charge-couple Device (CCD) camera and an excitation line using a He/Ne laser at 632.8 nm. Each spectrum was registered using a 10 s measurement time.

### 4.5. Drug Loading Study

The loading rate was calculated through the Thermo Gravimetry (TG) curve. The TG-DTA curve was studied using a simultaneous thermal analyzer (STA499C, NETZSCH, Selb, Germany). N_2_ was chosen as the heating environment and the temperature was decreased by 10 per minute. The drug load rate in the carrier can be estimated by Equation (1) according to the mass reduction at different temperatures and the heat absorption and exothermic peak at different temperatures.

(1) $$\mathrm{Drug}\;\mathrm{Loading}\;(w/w)=\frac{\mathrm{total}\;\mathrm{weight}\;\mathrm{loss}-\mathrm{material}\;\mathrm{weight}\;\mathrm{loss}}{1-\mathrm{material}\;\mathrm{weight}\;\mathrm{loss}}\times 100\%$$

### 4.6. In Vitro Drug Release Study

In order to evaluate the drug sustained-release behavior of drugs from RSA, the experiments were performed with resveratrol powder and RSA. The time dependence of resveratrol release from the drug carrier in two different solutions—simulated gastric fluid (pH = 2) and phosphate-buffered saline (pH = 7.4)—was investigated; the solutions simulated the conditions of gastric acid and body fluid, respectively. Both of the conditions did not involve protease. Resveratrol powder (15 mg) and RSA, with an equivalent dose of resveratrol, were added into a packet separately. The packet was immersed in a 500 mL solution and stirred under a paddle rotation speed of 100 rpm. The temperature was maintained at 37 °C. Three samples of 1 mL each were withdrawn at specific intervals (1 min, 5 min, 15 min, 30 min, 1 h, 2 h, 3 h, 4 h, 5 h, 6 h, 7 h, and 24 h), and the incubation solution was replenished with 1 mL of diluted ChP or PBS after each sampling. The resveratrol concentration was measured from the maximum absorbance at 308 nm with a UV spectrophotometer. Tests under the same conditions were carried out three times and the data were shown as mean ± SD. The release curve was drawn according to the average and standard deviation of three values at each moment. Regression analysis was used with the Higuchi model, zero-order model, and first-order model to reflect the release behavior of drug from RSA.

### 4.7. In Vitro Cytotoxicity Effect of RSA

The 3-(4,5-dimethylthiahiazo-2-y1)-2,5-diphenyltetrazolium bromide (MTT) cell viability assay was used to determine the survival rate of chondrocytes. Human normal chondrocytes (TC28a2) were used in the experiment, as well as 90% high glucose Dulbecco’s Modified Eagle Medium (DMEM)/F-12 and 10% fetal bovine serum (FBS), cultured in a 5% CO_2_ constant temperature incubator at 37 °C. The culture medium was changed every 2 days. When the cells grew to 80%–90% fusion, they were digested with 0.25% trypsin (containing 0.02% EDTA) and sub-cultured. The cells were inoculated in 96-well plates with 2.0 × 104 cells/well. The volume of each well was 100 μL. After 24 h of pre-culture, the cells were attached to the wall and grew. Three wells in each group were added with different concentrations of drugs and blank PBS control, respectively. After 24 h, 48 h, and 72 h of culture, 20 μL of MTT solution were added to each hole. After 4 h of incubation, the supernatant was discarded and 150 μL DMSO were added to each hole to terminate the reaction. The culture plate was shaken horizontally for 30 min, and the absorbance was measured at 490 nm with an enzyme-linked detector. The cell viability rate was then calculated. Statistical significance between different durations (24 h, 48 h, 72 h) was assessed using one-way ANOVA with Tukey’s multiple comparison tests (* *p* < 0.05, ** *p* < 0.01).

(2) $$\mathrm{Cell}\;\mathrm{Viability}\;\mathrm{Rate}\%=\frac{\mathrm{Absorbance}(\mathrm{sample},490\;\mathrm{nm})}{\mathrm{Absorbance}(\mathrm{contral},490\;\mathrm{nm})}\times 100\%$$

## 5. Conclusions

In this research, resveratrol-loaded of silica aerogel was successfully prepared through loading the drug before gel. The silica aerogel allows drugs such as resveratrol to preserve amorphous structure after loading on silica aerogels. As well, high drug loading rate (19%) was obtained. From the drug release test, more than 60% of resveratrol were observed releasing from RSA in the first 5 min which faster than pure powder. In the next 6 h, 20% of resveratrol was sustained release from RSA. Thus, compared to the crystalline form of the drug, slower drug release in the long-term procedure can be obtained. MTT assay and cell viability were performed on synthesized silica aerogel samples. It was found that the silica aerogels have a high potential for cell survival and have no cytotoxicity. Low biotoxicity makes it possible for pharmacists, in the future, to pay special attention to the widespread use of them in the drug carrier systems. In the future, in-vivo cytotoxicity study based on the tissue level will be carried out and animal models will be constructed to investigate the mechanism of RSA.

## Figures and Tables

**Table 1:** Correlation coefficients of three kinetic models applied to resveratrol released from RSA.

Kinetic Model	Pure Drug		RSA	
	R^2^ (PBS)	R^2^ (ChP)	R^2^ (PBS)	R^2^ (ChP)
Zero-order	0.995	0.999	0.639	0.701
First-order	0.809	0.950	0.974	0.985
Higuchi model	0.956	0.857	0.924	0.968
